# Evaluability assessment of the Brazilian National Food and Nutrition Policy, Fortaleza, Ceará, 2011-2022

**DOI:** 10.1590/S2237-96222026v35e20240768.en

**Published:** 2025-12-11

**Authors:** Amanda Gomes Queiroz Freitas, Maria Lara de Almeida Pinheiro, Lucas Silva Leite, Maria Socorro de Araújo Dias, Carlos André Moura Arruda, José Anael Neves, Cláudia Machado Coelho Souza de Vasconcelos

**Affiliations:** 1 Universidade Estadual do Ceará, Fortaleza, CE, Brazil Universidade Estadual do Ceará Fortaleza CE Brazil; 2 Universidade Estadual Vale do Acaraú, Sobral, CE, Brazil Universidade Estadual Vale do Acaraú Sobral CE Brazil

**Keywords:** Health Policy, Health Evaluation, Nutrition Programs and Policies, Nutrition Policy, Qualitative Research, Política de Salud, Evaluación en Salud, Programas y Políticas de Nutrición y Alimentación, Política Nutricional, Investigación Cualitativa.

## Abstract

**Objective:**

To conduct an evaluability assessment of the Brazilian National Food and Nutrition Policy in Fortaleza, Ceará, and to present the outcomes.

**Methods:**

This evaluability assessment was conducted between March 2023 and March 2024. A series of semi-structured interviews was conducted, and an analysis of legal, regulatory, and technical documents relating to the period from 2011 to 2022 was undertaken. The outcomes of the policy, according to key informants, were assessed through time trend analysis, with the calculation of the Annual Growth Rate (AGR) and its respective 95% confidence interval (95%CI), using the Prais-Winsten regression model.

**Results:**

A total of 24 key informants (managers, health professionals, and users) involved in the implementation of the policy participated in the study. The four components of the policy’s theoretical-logical model-food and nutrition surveillance; prevention of nutritional deficiency-related conditions; promotion of adequate and healthy eating; and comprehensive care for individuals with chronic diseases and/or special nutritional needs-were selected based on their relevance and recurrence in official documents and key informant interviews. Among the impacts attributed to the policy, the reduction in childhood malnutrition was particularly noteworthy, and the analysis of temporal trends revealed a decrease in severe thinness (AGR -4.94%; 95%CI -7.74; -2.05) in children aged 5 to 10 years, as well as an annual reduction of 4.94% in prevalence.

**Conclusion:**

The Brazilian National Food and Nutrition Policy is currently being implemented. The development of the theoretical-logical model has provided an opportunity to reflect on the challenges involved in its implementation, including internal and external contexts.

## Resumen

**Objetivo:**

Realizar una evaluación de evaluabilidad de la Política Nacional de Alimentación y Nutrición de Brasil en Fortaleza, Ceará, y presentar sus resultados.

**Métodos:**

Esta evaluación de evaluabilidad se llevó a cabo entre marzo de 2023 y marzo de 2024. Se realizaron entrevistas semiestructuradas y se analizó una serie de documentos legales, normativos y técnicos correspondientes al período de 2011 a 2022. Los resultados de la política, según informantes clave, fueron evaluados mediante análisis de tendencia temporal, calculando la Tasa de Crecimiento Anual (TCA) y su respectivo intervalo de confianza del 95% (IC95%), utilizando el modelo de regresión de Prais-Winsten.

**Resultados:**

Participaron en el estudio 24 informantes clave (gestores, profesionales de salud y usuarios) involucrados en la implementación de la política. Los cuatro componentes del modelo teórico-lógico de la política -vigilancia alimentaria y nutricional; prevención de condiciones relacionadas con deficiencias nutricionales; promoción de la alimentación adecuada y saludable; y atención integral a personas con enfermedades crónicas y/o necesidades nutricionales especiales- fueron seleccionados por su relevancia y recurrencia en los documentos oficiales y en las entrevistas con los informantes clave. Entre los impactos atribuidos a la política, se destacó la reducción de la desnutrición infantil, y el análisis de tendencias temporales reveló una disminución de la delgadez severa (TCA -4,94%; IC95% -7,74; -2,05) en niñas y niños de 5 a 10 años, así como una reducción anual del 4,94% en la prevalencia.

**Conclusión:**

La Política Nacional de Alimentación y Nutrición de Brasil se encuentra actualmente en proceso de implementación. La construcción del modelo teórico-lógico permitió reflexionar sobre los desafíos involucrados en su implementación, considerando tanto los contextos internos como externos.

**Ethical aspects**

This research respected ethical principles, having obtained the following approval data:

Research ethics committee: Universidade Estadual do Ceará

Opinion number: 6060195

Approval date: 15/5/2023

Certificate of submission for ethical appraisal: 68254323.1.0000.5534

Informed consent record? Obtained from all participants prior to data collection

## Introduction

The Brazilian National Food and Nutrition Policy (*Política Nacional de Alimentação e Nutrição* - PNAN), which was approved in 1999 and updated in 2011, aims to improve health and nutrition by promoting proper and healthy eating habits, food and nutrition monitoring, prevention, and comprehensive care for food and nutrition-related issues ^1^.

During its 25 years of implementation, notable breakthroughs have been made, including the publication of Brazilian dietary guidelines, the promotion of research into food and nutrition, the creation of a regulatory agenda, and the decentralization of financial resources for executing PNAN actions with the Financing of Food and Nutrition Actions (*Financiamento das Ações de Alimentação e Nutrição* - FAN) mechanism ^2^^,^^3^^,^^4^. 

The implementation of intersectoral food and nutrition programs and actions at the local level represents a significant challenge for addressing the multiple burdens of malnutrition ^2^. This approach presupposes the need for longitudinal and territorial care that considers the social determinants of health involved in the health-disease process. Therefore, it is at the local level that the policy is effectively implemented ^3^.

Actions to control nutritional disorders, such as iron and vitamin A deficiency, have historically been prioritized over actions to promote adequate and healthy eating and prevent other nutritional problems, which has gained further relevance with the new edition of the PNAN ^4^. Another study identified weaknesses in the implementation of the policy concerning social participation and control, funding, coverage, and use of the Food and Nutrition Surveillance System (*Sistema de Vigilância Alimentar e Nutricional* - SISVAN), strategies to address noncommunicable diseases, and the prevention of overweight, obesity, and nutritional deficiencies. Additionally, it identified gaps in the production of knowledge on the implementation of PNAN programs ^5^.

Public policy evaluation is an essential tool for understanding the effectiveness and efficiency of government actions. In the health sector, it plays a key role in qualifying care coordination and guiding decision-making. Several initiatives aimed at evaluating and improving Primary Health Care have already been developed in Brazil, including the National Program for Improving Access and Quality of Primary Health Care (*Programa de Melhoria do Acesso e Qualidade da Atenção Primária à Saúde* - PMAQ), which was discontinued during its third cycle, revealing a weak evaluation culture in primary health care management. Therefore, evaluation-oriented studies are relevant for understanding the implementation of programs and policy actions at the local level. In this context, evaluability assessments are included ^6^^,^^7^.

They serve as a pre-evaluation process based on a set of procedures designed to consider the interests of stakeholders and maximize the usefulness of the evaluation ^7^. This type of assessment creates a favorable environment for evaluation, seeks agreement among stakeholders on the interest and potential users of the evaluation, promotes shared understanding of the policy’s nature and objectives, and increases the likelihood of use of its findings ^7^^,^^8^. 

One of the key outputs of this type of study is the theoretical-logical model, a visual framework that outlines how the policy works and helps define the evaluation focus of the research (^7^^,^^9^^-^^11^). 

The relevance of conducting an evaluability assessment of PNAN at the municipal level lies in the absence of previous studies and in the potential of this policy to address the current challenging epidemiological scenario in Brazil. Accordingly, the objective of this study was to conduct an evaluability assessment of the Brazilian National Food and Nutrition Policy in Fortaleza, Ceará, and to present the outcomes.

## Methods

Study design

A qualitative exploratory-descriptive study was conducted as a pre-assessment of a public policy, with a focus on evaluating its feasibility and readiness for assessment. Research of this nature provides insight into the extent to which a policy is being implemented convergently to address the problem that prompted it and whether it can be evaluated ^7^^,^^8^. The study adopted the framework proposed by Leviton et al. ^11^. According to these authors, an evaluability assessment involves the following steps: 1) identifying the policy’s activities, objectives, and goals; 2) developing and validating the theoretical-logical model with key informants; 3) analyzing and comparing between the policy’s reality and the theoretical-logical model; 4) formulating recommendations and defining the evaluation questions ^10^^,^^11^. 

Setting

The study was conducted in Fortaleza, capital of the state of Ceará, with an estimated population of 2,428,678 inhabitants ^12^.

Study phases and instruments

In Phase 1 (March to November 2023), an analysis of official documents published between 2011 and 2022 was conducted. This period corresponds to the time elapsed between the last update of the Brazilian National Food and Nutrition Policy (*Política Nacional de Alimentação e Nutrição* - PNAN) and the start of the research. To gain a better understanding of the policy, we conducted a thorough and in-depth review of Brazilian federal legislation ^13^^-^^15^, as well as four management reports from the Ministry of Health’s General Coordination of Food and Nutrition (Virtual Health Library). 

Regarding Fortaleza’s municipal management, four local health plans, 11 annual health programs, four multi-year plans, one Fortaleza 2040 plan, 11 annual management reports, and 12 annual budget laws were reviewed. These documents were obtained from the official website of the city hall, under the tabs Transparency Portal and Access to Information, as well as from the website of the Municipal Health Council of Fortaleza. 

In Phase 2, the theoretical-logical model was developed and analyzed, aiming to clearly and coherently describe the functioning of the PNAN, based on both documents and interviews with key informants. The structure of the theoretical-logical model consisted of inputs, activities, outputs, outcomes, and impact ^7^. In addition, to test the hypotheses included in the model, a consistency test was applied using “if-then” statements among its elements. For example: “If these outputs are delivered, then intermediate outcomes will be achieved” ^16^. 

Semi-structured interviews were conducted from May to November 2023, using an interview guide developed by the researchers, with pre-tested questions based on Mendes et al. ^7^. These questions addressed knowledge of the policy, its funding, typical food and nutrition-related problems, programs and strategies implemented to address those issues, as well as facilitators and barriers to implementation. A total of 24 key informants participated in the interviews, including 14 health managers, six healthcare professionals, and four health council members, all with at least one year of experience in Primary Health Care. The information power principle was applied to determine the sample size ^17^. 

Data processing and analysis

Interviews were audio-recorded and later transcribed. A thorough reading and analysis were then conducted in order to identify perspectives on the policy’s objectives, the problems it seeks to address, implementation aspects, programs and actions, inputs, outcomes, strengths, weaknesses, and the need for evaluation. 

Regarding the impacts, it was decided that indicators reported during the interviews would be subjected to time trend analysis using the Prais-Winsten regression model. In this model, a p-value≥0.05 indicated a stable trend, and a p-value<0.05 indicated an increasing or decreasing trend, depending on whether the annual variation was positive or negative, respectively.

After completing the theoretical-logical model, a virtual meeting was held in December 2023 to review the model in an open discussion ^7^ with a committee of five experts: a nurse with expertise in the theoretical-logical model, a pedagogue with experience in primary health care management, and three nutritionists with expertise in PNAN. The discussion addressed logical relationships between the policy’s objectives, activities, and outcomes, as well as the model’s appearance, relevance, and consistency. The recommended adjustments were then made. 

With the revised theoretical-logical model, the key informants were once again consulted to validate it between December 2023 and March 2024. A printed version of the theoretical-logical model was used, along with a guide developed by the researchers containing the following questions: “What do you think of the way the theoretical-logical model looks?”; “Do you find the content of each column relevant?”; “Do you believe that this document (the theoretical-logical model) represents the situation of food and nutrition policy in Fortaleza?” and “Do you believe that this document reflects what was said in your interview?” Twenty key informants participated in this phase (a 16.67% loss compared to the previous one); four did not respond or were unavailable. The final adjustments to the model were then made ^16^. 

In Phase 3, the reality of the policy was analyzed and compared with the theoretical-logical model by consulting the field diary records of one of the researchers in this study, based on her visits to the study setting ^7^. Of particular note is the researcher’s experience as a nutritionist at the Family Health Support Center in the municipality from January 2017 to June 2019. This analysis helped verify whether all the elements mentioned by key informants or described in official policy documents were included in the model ^7^.

Finally, in Phase 4, the authors’ recommendations on the policy were formulated, and the evaluation questions were presented. The selection of the identified questions ^18^ will be conducted at a later stage.

## Results

The theoretical-logical model (Figure 1) was divided into four components: ^1^ food and nutrition surveillance; ^2^ prevention of conditions related to nutritional deficiencies; ^3^ promotion of adequate and healthy eating; ^4^ comprehensive care for individuals with chronic diseases and/or special nutritional needs. Although the Brazilian National Food and Nutrition Policy comprises nine guidelines, four components were listed in the model, as they most robustly represent the scope of policy implementation in Fortaleza. 

Internally, PNAN is part of the Primary and Psychosocial Care Coordination Unit, indicating the connection between the program and primary health care (Figure 1). 

Both official documents and key informant interviews revealed that the policy addresses the double burden of malnutrition. However, not all informants explicitly mentioned both aspects of the issue (i.e., undernutrition/nutrient deficiencies and overweight/chronic diseases). 

Food and nutrition initiatives in Fortaleza cut across primary health care work processes. Food and nutrition initiatives in Fortaleza cut across primary healthcare processes. Family health and e-Multi teams distinguish between food and nutrition monitoring and food and nutrition guidance/education in individual care (childcare, prenatal care, elderly care, diabetic care, hypertensive care, Bolsa Família Program [BFP], *Cresça com Seu Filho* [Grow with Your Child Program], home visits) and in collective activities (educational groups, *Saúde na Escola* [Health at School Program], and *Gente Adolescente* [Teenage People Program]), in addition to micronutrient supplementation and training. Some of Fortaleza’s own programs are worth mentioning: *Gente Adolescente* [Teenage People Program], which is similar to *Saúde na Escola* [Health at School Program] except for the age group of the target audience, *Unidade Amiga da Primeira Infância* [Early Childhood Friendly Unit], and *Cresça com Seu Filho* [Grow with Your Child Program]. 

With regard to component 1 of the theoretical-logical model (Figure 1), it is worth noting the predominance of activities, outputs, and outcomes related to monitoring the weight and height of BFP beneficiaries, although this is also mentioned in routine childcare and prenatal care. For other groups, there is either incomplete data entry or incorrect recording.

Thus, food and nutrition surveillance is only partially implemented, as it fails to provide a population-level nutritional profile and instead focuses on priority groups only.

Within component 2, that is, prevention of conditions related to nutritional deficiencies, actions related to the national programs for iron supplementation (Programa Nacional de Suplementação de Ferro - PNSF) and vitamin A supplementation (Programa Nacional de Suplementação de Vitamina A - PNVITA) were identified: prescription of supplements, inventory control, and dietary guidance during childcare and prenatal care appointments. However, no data on coverage were found, and interviewees also reported a lack of training to implement the programs. Therefore, this component was also considered partially implemented. 

In component 3, promotion of adequate and healthy eating, it was evident that, as part of the team’s routine individual care (prenatal, childcare, older adults, and people with chronic diseases), guidance and education on diet and nutrition predominated, in addition to, less frequently, collective initiatives under the *Saúde na Escola* Program, *Gente Adolescente* Program, and other educational groups, when available. Although present in both individual and group formats, professionals reported lacking training to address this topic. No other strategies related to this component were identified, such as actions to change the food environment. As a result, this component is also partially implemented.

Component 4, comprehensive care for individuals with chronic diseases and/or special nutritional needs, appeared to be prioritized. Individual and home care predominates for patients with diabetes and/or hypertension, bedridden individuals, and those with special dietary needs, even though there are no established lines of care or care flows for food and nutrition. This component was also considered partially implemented.

As for policy-related impacts, the decision was made to include in the theoretical-logical model only those mentioned by interviewees and corroborated by the analysis of temporal trends in indicators (2011-2022). The analysis revealed a downward trend in severe thinness among children aged 5 to 10 years (APC -4.94%; 95%CI -7.74; -2.05), indicating an annual 4.94% decrease in prevalence. 

**Figure 1:**
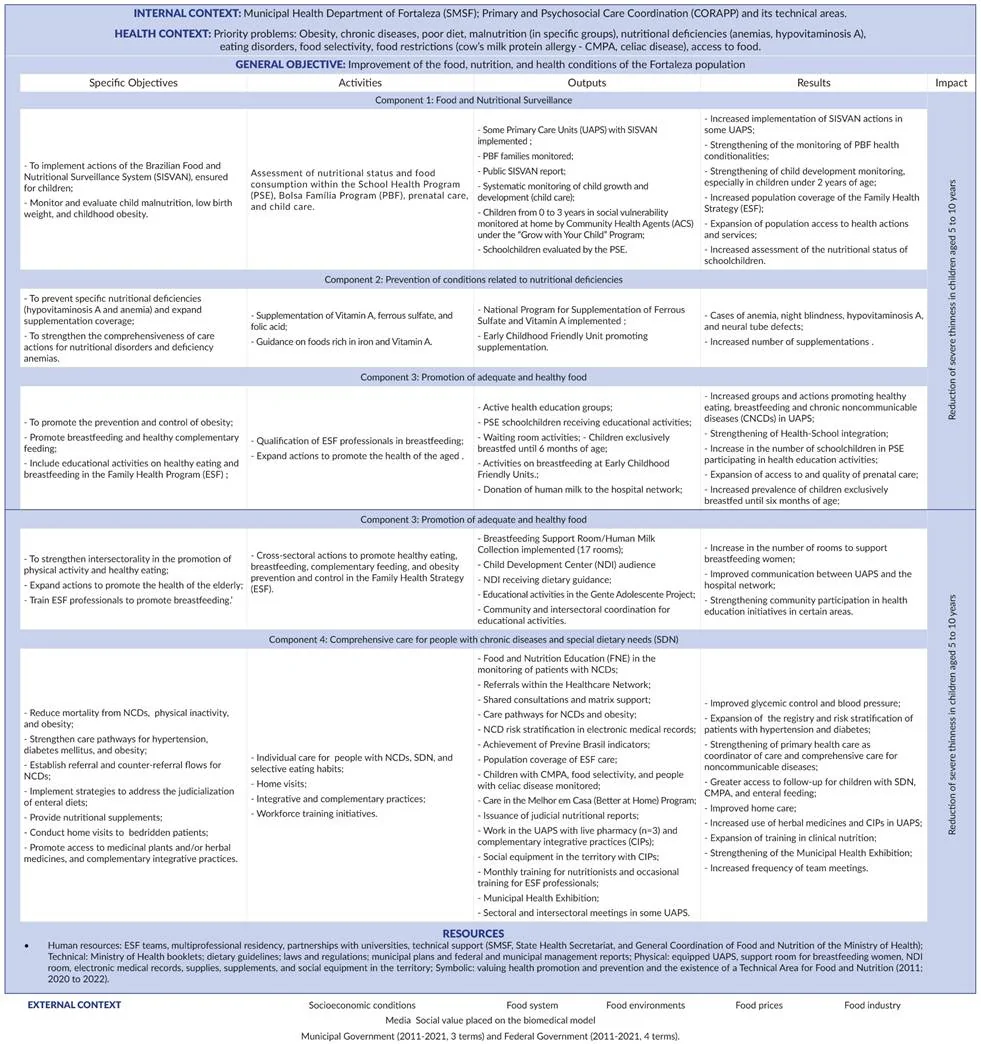
Theoretical-logical model of the Brazilian National Food and Nutrition Policy. Fortaleza, 2024

Table 1 outlines the strengths and weaknesses of PNAN implementation in Fortaleza, according to key informants. 

Among the weaknesses in policy implementation, the most notable are the excessive workload and precarious employment conditions of primary care teams, which undermine continuity and relationships within care. There is an evident shortage of nutritionists in health units and management, who are equally overburdened. The inclusion of more nutritionists in the network was mentioned as a critical need, given the added value they bring to the food and nutrition agenda. 

With regard to financial resources, documents identified federal funding sources (e.g., Financing of Food and Nutrition Actions, *Saúde na Escola* Program, Family Health Support Center/Multiprofessional Teams (e-Multi), and COVID-19-specific ordinances) and municipal sources (e.g., annual budget laws). Nonetheless, the municipal resources allocated in the annual budget laws were not mentioned in the interviews, and the analysis of annual management reports suggests that this budget item was not implemented during the analyzed period. Moreover, there was no indication of state-level funding allocated for this purpose in the municipality.

**Table 1:** Strengths and weaknesses of implementing the Brazilian National Food and Nutrition Policy according to managers, health professionals, and users. Fortaleza, 2024 (n=24)

Weaknesses	Strengths
Little or no knowledge of the policy among managers, health professionals, and users. Difficulty in managing and low visibility of the Brazilian National Food and Nutrition Policy. Low number of health professionals, especially nutritionists, in care and management, work overload, staff turnover, and weak employment relationships. Continuity of care and weak ties between professionals and users. Non-recognition of non-nutritionist health professionals as subjects of the Brazilian National Food and Nutrition Policy. Absence of an institutionalized technical area for food and nutrition. Incipient intersectorality. Prioritization of the biomedical model. Insufficient team meetings and matrix support. Filling in anthropometric data for the Food and Nutrition Surveillance System focused on beneficiaries of the Bolsa Família Program. Absence of nutrition protocols and care flows for educational activities and care. Collective food and nutrition education actions occur predominantly through academic, sporadic, and isolated initiatives. Insufficiency or absence of human, physical, and financial resources. Weak management of financial resources. Violence hinders the population’s access to health units and professionals in the territories.	*Gente Adolescente* [Teenage People] Project, *Saúde na Escola* [Health at School] Program, Breastfeeding Support Room, *Conhecer para Acolher* [Knowing to Welcome] Project, *Unidade Amiga da Primeira Infância* [Early Childhood Friendly Unit], *Núcleo de Desenvolvimento Infantil* [Child Development Center], Bolsa Família Program, *Previne Brasil* [Prevent, Brazil] (goals related to the health of pregnant women and children). Social facilities such as community centers, the Social Assistance Reference Center, restaurants for low-income individuals, and community kitchens. Multidisciplinary Home Care Teams, e-Multi team, Family Health Strategy, Community Health Agent. Health education, operational groups, discussion groups, waiting rooms, and active search. TV sets in some health facilities. Teamwork and good relationships between professionals. Support from municipal management for food and nutrition initiatives, when proposed. Decentralized technical support from regional health authorities for initiatives linked to the Brazilian National Food and Nutrition Policy. Potential for intersectoral collaboration between health, education, social assistance, and other sectors. Coordination between municipal and state management. Presence of higher education institutions in the services. Nutritionist involvement in the treatment of obesity. Cross-system information sharing on nutritional status and food consumption. Financial incentives linked to indicators and actions (e.g., teenagers’ health).

## Discussion 

The Brazilian National Food and Nutrition Policy (PNAN) was partially implemented in Fortaleza during the period analyzed. 

Despite more than 25 years of existence, we found no evaluability assessment studies of the policy in the literature. Instead, only evaluations of programs linked to it were found ^19^^-^^21^, even though studies of this nature have been conducted in the health field ^22^. 

The municipality does not have its own food and nutrition policy, nor does the state government. Having a specific policy would better meet local needs and promote more effective decentralization ^23^^,^^24^. Thus, the analysis presented in this study was guided by the national policy. 

Documentary analysis revealed that the PNAN is well established at the federal level, which represents institutional progress. Nevertheless, the enactment of legal norms does not guarantee their effectiveness, which requires monitoring for their implementation ^25^.

The PNAN holds a secondary position in relation to other policies ^26^. Insufficient financial resources from the federal government are cited as a limiting factor in meeting the demands of the food and nutrition agenda. At the same time, central management acknowledges the need for greater municipal funding for this purpose. Challenges in financing food and nutrition initiatives have already been reported ^4^. 

The partial implementation of the components of the theoretical-logical model has consequences for the organization of nutritional care. The underuse of food and nutrition surveillance makes it impossible to diagnose the population, which is necessary for all stages of the life cycle ^23^^,^^27^. Food and nutrition surveillance restricted to Bolsa Família Program beneficiaries indicates a bureaucratic view of health requirements, exclusively for the fulfillment of management goals. Lack of knowledge and low appreciation of food and nutrition surveillance as a component of health care were also found in another study ^28^. 

Although actions related to micronutrient supplementation programs have been reported, coverage is low, according to public reports from the Food and Nutrition Surveillance System for Fortaleza ^29^, a recurring reality in other national scenarios ^30^^,^^31^. 

Despite the importance of promoting adequate and healthy eating in the PNAN, the situation in Fortaleza is similar to that nationwide, with priority given to measures to control nutritional problems at the expense of measures to promote adequate and healthy eating ^4^^,^^32^. The publication of dietary guidelines with national recommendations represents a step forward in promoting adequate and healthy eating. However, strategies are still needed to disseminate the messages contained in these guidelines ^27^. 

Comprehensive care for people with chronic diseases and special nutritional needs is prioritized through individual and home care, reflecting the persistence of the hegemony of the biomedical model in health ^28^^,^^33^. Added to this scenario is the overload of activities of the teams and the lack of material resources, which impacts health promotion actions. 

The weaknesses in policy implementation at the municipal level may be related to the absence of an institutionalized technical area for food and nutrition at the central level to support regional health technicians more systematically, as identified in studies with other scenarios ^24^^,^^34^. 

Intersectorality is viewed both as a weakness and a strength. Its strength is seen in actions associated with the Saúde na Escola (Health at School) Program, Gente Adolescente (Teenage People) Program, and Bolsa Família Program, but these are isolated cases. In addition to education, some partnerships with social assistance were mentioned within the scope of food and nutrition security, but these were also sporadic. It is important to expand intersectoral coordination so that the food and nutrition agenda is included in the definition and planning of other sectors ^35^, strengthening structural and synergistic public policies focused on the main nutritional problems ^20^.

As for the policy’s impact, the reduction in severe thinness among children aged 5 to 10 years reflects the implementation of PNAN in Fortaleza, despite the strong influence of external factors on this outcome.

Among the study’s limitations, it should be noted that the theoretical-logical model validation occurred individually and did not allow for negotiation among key informants regarding potential disagreements. However, the proposal for validation in workshops was not accepted by primary health care management due to losses related to the departure of professionals from services with high daily demand. Furthermore, the fact that the study originated from academic demand rather than from the health service may influence future developments and evaluations.

The evaluability assessment contributed to a better understanding of PNAN in Fortaleza, from its institutionalization to its day-to-day implementation. It also engaged key informants from different areas and led to the development of the logic model. 

Several challenges in implementing the policy were identified. Among them, it is noteworthy that key informants know little about it, despite their desire to strengthen it. It should be noted that the four components that comprise the theoretical-logical model represent, in a more robust manner, the scope of the policy’s implementation in the municipality. 

It can be concluded that PNAN is suitable for evaluation, as it was possible to characterize its key components, thereby providing input for an assessment aligned with the interests of the study participants. The following recommendations are presented for the implementation of the PNAN: invest in the policy as a whole, not just in specific actions focused on priority groups; include more policy indicators in the agreements of the Brazilian National Health System and make them visible to professionals, managers, and social control; periodically review the theoretical-logical model of the policy; disseminate the results of this research to managers, technicians, family health strategy professionals, and social control; conduct greater dissemination and training on the policy; promote municipal funding for the food and nutrition agenda; enable greater involvement of nutritionists in e-Multi teams; institutionally create a technical area for food and nutrition with responsibilities focused solely on this portfolio and/or with an expanded technical team; conduct evaluative studies for all the components described. 

The theoretical-logical model developed by this research portrays a framework that can serve as a reference to assist those involved in policy implementation, enabling its review through a participatory process ^10^. The model developed in this research can be studied and revised at any time, based on new guidelines, innovations in implementation strategies, or other analytical and/or evaluative perspectives. 
