# Supplementary material for: Evaluability assessment of the Brazilian National Food and Nutrition Policy, Fortaleza, Ceará, 2011-2022
Source: Epidemiol Serv Saude. 2025 Dec 11;35:20242407. doi: 10.1590/S2237-96222026v35e20240768.en (PMC13686297; doi:10.1590/S2237-96222026v35e20240768.en)
Supplement: Translation

# Translation: Estudo de avaliabilidade da Política Nacional de Alimentação e Nutrição, Fortaleza, Ceará, 2011-2022

**Authors:** Amanda Gomes Queiroz Freitas, Maria Lara de Almeida Pinheiro, Lucas Silva Leite, Maria Socorro de Araújo Dias, Carlos André Moura Arruda, José Anael Neves, Cláudia Machado Coelho Souza de Vasconcelos

**Affiliations:** Universidade Estadual do Ceará, Fortaleza, CE, Brasil Universidade Estadual do Ceará Fortaleza CE Brasil; 2 Universidade Estadual Vale do Acaraú, Sobral, CE, Brasil

**Keywords:** Política de Saúde, Avaliação em Saúde, Programas e Políticas de Nutrição e Alimentação, Política Nutricional, Pesquisa Qualitativa

## Abstract

**Objetivo:**

Empreender um estudo de avaliabilidade da Política Nacional de Alimentação e Nutrição em Fortaleza, Ceará, e apresentar seus resultados.

**Métodos:**

Estudo de avaliabilidade realizado entre março de 2023 e março de 2024. Realizou-se entrevistas semiestruturadas e análise de documentos legais, normativos e técnicos relativos ao período de 2011 a 2022. Os resultados da política, segundo os informantes-chave, foram analisados por meio de tendência temporal, calculando-se a Taxa de Incremento Anual (TIA) e seu respectivo intervalo de confiança (IC95%), usando o modelo de regressão de Prais-Winsten.

**Resultados:**

Participaram do estudo 24 informantes-chave (gestores, profissionais de saúde e usuários) envolvidos com a execução da política. Os quatro componentes do modelo teórico-lógico da política (vigilância alimentar e nutricional, prevenção de agravos relacionados às carências nutricionais, promoção da alimentação adequada e saudável e cuidado integral da pessoa com doenças crônicas e/ou necessidades nutricionais especiais) foram selecionados devido à sua relevância e à sua recorrência nas publicações oficiais e entrevistas com informantes-chaves. Dentre os impactos atribuídos à política, destacou-se a redução da desnutrição em crianças, e a análise de tendência temporal revelou redução da magreza acentuada (TIA -4,94%; IC95% -7,74; -2,05) nas crianças de 5 a 10 anos, bem como uma redução anual de 4,94% na prevalência.

**Conclusão:**

A Política Nacional de Alimentação e Nutrição está em processo de implementação. A construção do modelo teórico-lógico permitiu refletir sobre os desafios na sua implementação, incluindo os contextos interno e externo.

Aspectos éticos

Esta pesquisa respeitou os princípios éticos, obtendo os seguintes dados de aprovação:

Comitê de ética em pesquisa: Universidade Estadual do Ceará

Número do parecer: 6.060.195

Data de aprovação: 15/5/2023

Certificado de apresentação de apreciação ética: 68254323.1.0000.5534

Registro do consentimento livre e esclarecido: Obtido de todos os participantes antes da coleta

## Introdução

A Política Nacional de Alimentação e Nutrição (PNAN), aprovada no ano de 1999 e atualizada em 2011, visa melhorar as condições de saúde e de nutrição, mediante a promoção de práticas alimentares adequadas e saudáveis, vigilância alimentar e nutricional, prevenção e cuidado integral aos agravos relacionados à alimentação e à nutrição ^1^.

Em seus 25 anos de implementação, destacam-se avanços como a publicação dos guias alimentares brasileiros, o fomento à pesquisa em alimentação e em nutrição, a construção de uma agenda regulatória e a descentralização de recursos financeiros para execução das ações da PNAN por meio do Financiamento das Ações de Alimentação e Nutrição (FAN) ^2^^,^^3^^,^^4^. 

A implementação de programas e de ações de alimentação e de nutrição de caráter intersetorial em âmbito local constitui um importante desafio para o enfrentamento da múltipla carga da má nutrição ^2^. Pressupõe-se a necessidade de um cuidado longitudinal e territorial que considere os determinantes sociais envolvidos no processo saúde-doença. Portanto, é no contexto local que a política se efetiva ^3^.

As ações de controle dos agravos nutricionais, como deficiência de ferro e de vitamina A, foram priorizadas, historicamente, em relação às ações de promoção da alimentação adequada e saudável e de prevenção de outros problemas nutricionais, o que ganhou mais relevância com a nova edição da PNAN ^4^. Outro estudo constatou fragilidades na implementação da política no que concerne: à participação e ao controle social; ao financiamento; à cobertura e à utilização do Sistema de Vigilância Alimentar e Nutricional; às estratégias para o enfrentamento das doenças crônicas não transmissíveis; e à prevenção do sobrepeso, da obesidade e das carências nutricionais. Além de ter verificado lacunas na produção de conhecimento sobre a implantação de programas da PNAN ^5^.

A avaliação de políticas públicas é uma ferramenta essencial para entender a eficácia e a eficiência das ações governamentais. No contexto da saúde, ela é fundamental para a qualificação da coordenação do cuidado e para tomadas de decisão. Muitas iniciativas voltadas para avaliação e aperfeiçoamento da Atenção Primária à Saúde (APS) já foram desenvolvidas no Brasil, entre elas, o Programa de Melhoria do Acesso e Qualidade da Atenção Primária à Saúde (PMAQ), descontinuado em seu terceiro ciclo, evidenciando uma frágil cultura avaliativa na gestão da APS. Portanto, se faz relevante a realização de estudos de cunho avaliativo para compreender a implementação dos programas e das ações das políticas a nível local. Nesse escopo, incluem-se os estudos de avaliabilidade ^6^^,^^7^.

Os estudos de avaliabilidade constituem-se como uma pré-avaliação sustentada em um conjunto de procedimentos de modo que os interesses dos envolvidos são considerados para maximizar a utilidade da avaliação ^7^. Esse tipo de estudo proporciona um ambiente favorável para avaliação, busca a concordância quanto ao interesse na realização da avaliação e os possíveis usuários do estudo, constrói entendimento entre os envolvidos sobre a natureza e os objetivos da política e aumenta as possibilidades de utilização dos resultados da avaliação ^7^^,^^8^. 

Um dos produtos desse tipo de estudo é o modelo teórico-lógico, um esquema visual que expõe o funcionamento da política e ajuda a apresentar o foco avaliativo da pesquisa ^7^^,^^9^^-^^11^. 

A relevância de um estudo de avaliabilidade da PNAN no âmbito municipal deve-se à ausência de estudos anteriores e à potência dessa política frente ao desafiador cenário epidemiológico atual no Brasil. Diante disso, o objetivo desta pesquisa foi empreender um estudo de avaliabilidade da Política Nacional de Alimentação e Nutrição em Fortaleza, Ceará, e apresentar seus resultados.

## Métodos

Desenho do estudo

Estudo de pré-avaliação de política pública, do tipo avaliabilidade, sustentado na abordagem qualitativa de natureza exploratória-descritiva. Pesquisas com esse design permitem reconhecer em que medida uma política está sendo implementada em convergência com o propósito de responder ao problema que a originou e se há condições de ser avaliada. ^7^^,^^8^. Optou-se pelo referencial de Leviton et al. ^11^. Para esses autores, um estudo de avaliabilidade consiste nas seguintes etapas: 1) identificação de atividades, objetivos e metas da política; 2) desenvolvimento e pactuação do modelo teórico-lógico com os informantes-chave; 3) análise e comparação entre a realidade da política e o modelo teórico-lógico; 4) elaboração de recomendações e definição de perguntas avaliativas ^10^^,^^11^. 

Cenário do estudo

O cenário do estudo foi Fortaleza, capital do Ceará, com população estimada em 2.428.678 milhões de habitantes ^12^.

Etapas do estudo e instrumental

Na etapa 1, entre março e novembro de 2023, realizou-se análise de documentos oficiais publicados entre os anos de 2011 e 2022. O período de análise transcorreu entre a última atualização da Política Nacional de Alimentação e Nutrição (PNAN) até a data de início da pesquisa. Para aproximação e entendimento da política, procedeu-se à leitura exaustiva e em profundidade da legislação federal ^13^^-^^15^, além de quatro relatórios de gestão da Coordenação Geral de Alimentação e Nutrição do Ministério da Saúde (Biblioteca Virtual em Saúde). 

Da gestão municipal, foram lidos quatro planos municipais de saúde, 11 programações anuais de saúde, quatro planos plurianuais de Fortaleza, um plano Fortaleza 2040, 11 relatórios anuais de gestão e 12 leis orçamentárias anuais. Esses documentos foram obtidos no site oficial da prefeitura, nas abas Portal da transparência e Acesso à informação, além do sítio eletrônico do Conselho Municipal de Saúde de Fortaleza. 

Na etapa 2, foi desenvolvido e analisado o modelo teórico-lógico, com o objetivo de descrever de maneira clara e coerente o funcionamento da PNAN, baseando-se nos documentos e em entrevistas com informantes-chave. A estrutura do modelo teórico-lógico foi composta de recursos, atividades, produtos, resultados e impacto ^7^. Além disso, para analisar as hipóteses elencadas no modelo, aplicou-se o teste de consistência usando a assertiva “se-então” nos seus elementos. Por exemplo: “Se tais produtos são realizados, então alcanço os resultados intermediários” ^16^. 

As entrevistas semiestruturadas ocorreram entre maio e novembro de 2023 e foram realizadas por meio de roteiro de elaboração própria, com questões previamente testadas construídas segundo Mendes et al. ^7^, as quais versavam sobre o conhecimento da política, seu financiamento, problemas de alimentação e nutrição mais frequentes, ações, programas e estratégias utilizadas para enfrentamento dos principais problemas de alimentação e de nutrição, além de aspectos facilitadores e barreiras para implementação. Participaram das entrevistas 24 informantes-chave, sendo 14 gestores, seis profissionais de saúde e quatro conselheiros de saúde, com experiência mínima de um ano na Atenção Primária à Saúde (APS). O princípio de information power foi aplicado para o fechamento amostral ^17^. 

Tratamento e análise dos dados

As entrevistas foram gravadas em áudio e posteriormente transcritas. Em seguida, procedeu-se à leitura-análise exaustiva, a fim de compreender concepções sobre objetivos, problemas que a política pretende resolver, implementação, programas e ações, recursos, resultados, potencialidades, fragilidades e necessidade de avaliação. 

No tocante aos impactos, decidiu-se que aqueles reportados nas entrevistas teriam seus indicadores submetidos à análise de tendência temporal usando o modelo de regressão de Prais-Winsten, em que: p-valor≥0,05 indicaram tendência de estabilidade, e p-valor<0,05, tendência crescente ou decrescente, conforme a variação anual positiva ou negativa, respectivamente.

Após a finalização do modelo teórico-lógico, realizou-se uma reunião virtual, em dezembro de 2023, para apreciação do modelo por meio de uma discussão aberta ^7^ com um comitê de cinco especialistas: uma enfermeira com expertise em modelo teórico-lógico, um pedagogo com expertise na gestão da APS e três nutricionistas com expertise na PNAN. Discutiu-se sobre as relações de lógica entre os objetivos, atividades e resultados da política, bem como sobre aparência, pertinência e consistência do modelo. Em seguida, realizaram-se os ajustes recomendados. 

Com o modelo teórico-lógico ajustado, os informantes-chave o apreciaram novamente e validaram o modelo, entre dezembro de 2023 e março de 2024. Utilizou-se a versão impressa do modelo teórico-lógico e um roteiro elaborado pelas pesquisadoras com as seguintes perguntas: “O que você acha da aparência do modelo teórico-lógico?”; “Você acha o conteúdo de cada coluna pertinente?”; “Você acredita que esse documento (o modelo teórico-lógico) representa a situação da política de alimentação e de nutrição em Fortaleza?”; e “Você acredita que esse documento contempla o que foi dito na sua entrevista?”. Participaram dessa etapa 20 informantes-chave (uma perda de 16,67% em relação à etapa anterior); quatro não responderam aos contatos ou não tiveram disponibilidade. Posteriormente, realizaram-se os ajustes finais no modelo ^16^. 

Na etapa 3, procedeu-se à análise e à comparação entre a realidade da política e o modelo teórico-lógico mediante consultas aos registros do diário de campo de uma das pesquisadoras deste estudo decorrentes das suas visitas ao cenário do estudo ^7^. Destaca-se a experiência da pesquisadora como nutricionista do Núcleo de Apoio à Saúde da Família do município de janeiro de 2017 a junho de 2019. Essa análise permitiu identificar se todos os elementos referidos pelos informantes-chave ou descritos nos documentos oficiais da política foram considerados no modelo ^7^.

Por fim, na etapa 4, foram elaboradas as recomendações dos autores sobre a política e apresentadas as perguntas avaliativas. A fase de seleção das perguntas identificadas ^18^ será realizada posteriormente.

## Resultados

O modelo teórico-lógico (Figura 1) foi dividido em quatro componentes: ^1^ vigilância alimentar e nutricional; ^2^ prevenção de agravos relacionados às carências nutricionais; ^3^ promoção da alimentação adequada e saudável; e ^4^ cuidado integral da pessoa com doenças crônicas e de necessidades nutricionais especiais. Embora a Política Nacional de Alimentação e Nutrição (PNAN) possua nove diretrizes, foram elencados quatro componentes no modelo, por representarem de forma mais robusta o alcance da implementação da política em Fortaleza. 

No âmbito do contexto interno, a PNAN está na Coordenadoria de Atenção Primária e Psicossocial, refletindo a vinculação dela com a Atenção Primária à Saúde (APS) (Figura 1). 

Evidenciou-se, tanto nos documentos oficiais quanto nas entrevistas, a dupla carga de má nutrição como objeto de intervenção da política, ainda que nem sempre os informantes-chave mencionem os dois aspectos do problema (desnutrição/carências nutricionais e obesidade/doenças crônicas). 

As ações de alimentação e de nutrição em Fortaleza são transversais aos processos de trabalho da APS. Nesta, distinguem-se, pelas equipes de saúde da família e e-Multi, as ações de vigilância alimentar e nutricional e de orientação/educação alimentar e nutricional nos atendimentos individuais (puericultura, pré-natal, idoso, diabético, hipertensos, Programa Bolsa Família [PBF], Cresça com Seu Filho, visita domiciliar) e nas atividades coletivas (grupos educativos, Programa Saúde na Escola e Gente Adolescente), além de suplementação de micronutrientes e de capacitações. Destacam-se programas próprios do município: o Programa Gente Adolescente, semelhante ao Programa Saúde na Escola (PSE), exceto pela faixa etária do público-alvo; a Unidade Amiga da Primeira Infância; e o Programa Cresça com seu Filho. 

No tocante ao componente 01 do modelo teórico-lógico (Figura 1), ressalta-se a predominância de atividades, produtos e resultados relacionados ao acompanhamento de peso e altura dos beneficiários do PBF, embora seja mencionado na rotina da puericultura e no pré-natal. Para os demais públicos, há subpreenchimento ou preenchimento errôneo.

Logo, a vigilância alimentar e nutricional apresentou funcionamento parcial, pois não permite traçar um perfil alimentar e nutricional populacional, mas apenas de públicos prioritários.

No componente 02, prevenção dos agravos relacionados às carências nutricionais, foram identificadas ações relacionadas aos programas nacionais de suplementação de ferro (PNSF) e de vitamina A (PNVITA): prescrição da suplementação; controle de estoque; e orientações alimentares nos atendimentos de puericultura e pré-natal. Não foram localizados dados de cobertura, e os entrevistados também referiram ausência de capacitação para operacionalização dos programas. Assim sendo, infere-se que esse componente também está em funcionamento parcial. 

No componente 03, promoção da alimentação adequada e saudável, evidenciou-se, como parte dos atendimentos individuais de rotina da equipe (pré-natal, puericultura, idosos e portadores de doenças crônicas), a predominância de ações de orientação e de educação alimentar e nutricional, além de, em menor frequência, as ações coletivas do PSE, do Gente Adolescente e de demais grupos educativos, quando existentes. Mesmo presente nos atendimentos individuais e nas ações coletivas, os profissionais relataram que não há formação para atuação nessa temática e não foram identificadas outras estratégias relacionadas a esse componente, como ações dirigidas a mudanças no ambiente alimentar. Dessa forma, o componente 03 também está em implementação parcial.

Observou-se uma priorização do componente 04, cuidado integral da pessoa com doenças crônicas e de necessidades nutricionais especiais. Predominam os atendimentos individuais e domiciliares junto aos pacientes com diabetes e/ou com hipertensão, aos pacientes acamados e aos com necessidades alimentares especiais, ainda que não existam linhas de cuidado e fluxos de atendimento em alimentação e em nutrição estabelecidos. O componente 04 também está em implementação parcial.

Quanto aos impactos relacionados à política, decidiu-se incluir, no modelo teórico-lógico, somente aqueles citados pelos entrevistados e corroborados pela análise de tendência temporal de indicadores (2011-2022) realizada. A análise revelou tendência temporal de redução para magreza acentuada (TIA -4,94%; IC95% -7,74; -2,05) nas crianças de 5 a 10 anos, uma redução anual de 4,94% na sua prevalência. 

**Figura 1:** Modelo teórico lógico da Política Nacional de Alimentação e Nutrição. Fortaleza, 2024.

A Tabela 1 sinaliza potencialidades e fragilidades na implementação da PNAN no município segundo os informantes-chave. 

Dentre as fragilidades na implementação da política, destacam-se a sobrecarga de trabalho e os vínculos trabalhistas precários das equipes da APS, o que prejudica a longitudinalidade e os vínculos no cuidado. Evidencia-se o pequeno número de nutricionistas nas unidades de saúde e na gestão, igualmente sobrecarregados. Há demanda pela inclusão de mais nutricionistas na rede devido ao diferencial que esse profissional faz na agenda de alimentação e de nutrição. 

No tocante aos recursos financeiros disponíveis, observaram-se fontes federais (Financiamento das Ações de Alimentação e Nutrição; Programa Saúde na Escola; Núcleo de Apoio à Saúde da Família/e-Multi; e portarias específicas da pandemia) e municipais (leis orçamentárias anuais) nos documentos. No entanto, os recursos municipais previstos nas leis orçamentárias anuais não emergem das entrevistas, e a análise dos relatórios anuais de gestão indica que essa rubrica não foi executada no período analisado. Destaca-se que não existiu menção a recurso estadual com essa finalidade para o município.

**Tabela 1:** Potencialidades e fragilidades da implementação da Política Nacional de Alimentação e Nutrição segundo gestores, profissionais de saúde e usuários. Fortaleza, 2024 (n=24)

Fragilidades	Potencialidades
Pouco conhecimento ou desconhecimento da política pelos gestores, profissionais de saúde e usuários. Dificuldade de gestão e pouca visibilidade da Política Nacional de Alimentação e Nutrição. . Baixo quantitativo de profissionais de saúde, especialmente de nutricionistas, na assistência e na gestão, sobrecarga de trabalho, rotatividade de profissionais e vínculos trabalhistas fragilizados. Longitudinalidade do cuidado e vínculo dos profissionais com os usuários prejudicados. Não reconhecimento dos próprios profissionais de saúde não nutricionistas como sujeitos da Política Nacional de Alimentação e Nutrição. . Ausência de área técnica de alimentação e nutrição institucionalizada. Intersetorialidade incipiente. Priorização do modelo biomédico. Pouca realização das reuniões de equipe e matriciamento. Preenchimento dos dados antropométricos para o Sistema de Vigilância Alimentar e Nutricional focado em beneficiários do Programa Bolsa Família. Ausência de protocolos de nutrição e fluxos de atendimento para as atividades educativas e atendimentos. Ações de educação alimentar e nutricional coletivas ocorrem predominantemente por iniciativas acadêmicas, pontuais e isoladas. Insuficiência ou ausência de recursos humanos, físicos e financeiros. Gestão fragilizada dos recursos financeiros. Violência prejudica o acesso da população às unidades de saúde e dos profissionais nos territórios.	Projeto Gente Adolescente, Programa Saúde na Escola, Sala de Apoio à Amamentação, Projeto Conhecer para Acolher, Unidade Amiga da Primeira Infância, Núcleo de Desenvolvimento Infantil, Programa Bolsa Família, Previne Brasil (metas relacionadas à saúde da gestante e da criança). Equipamentos sociais como centros comunitários, Centro de Referência da Assistência Social, restaurantes populares e cozinhas comunitárias. Equipes Multiprofissionais de Atenção Domiciliar, equipe e-multi, Estratégia Saúde da Família, Agente Comunitário de Saúde. Educação em saúde, grupos operativos, rodas de conversa, salas de espera e busca ativa. Aparelhos de TV em algumas unidades de saúde. Trabalho em equipe e a boa relação entre profissionais. Apoio da gestão municipal para ações de alimentação e nutrição, quando propostas. Apoio técnico descentralizado por regionais de saúde para ações vinculadas à Política Nacional de Alimentação e Nutrição. Intersetorialidade potencial entre saúde, educação, assistência social e outros setores. Articulação entre gestão municipal e estadual. Presença das Instituições de Ensino Superior nos serviços. Atuação do nutricionista no tratamento da obesidade. Migração das informações sobre estado nutricional e consumo alimentar entre sistemas. Incentivo financeiro vinculado aos indicadores e ações (exemplo: saúde do adolescente).

## Discussão 

A Política Nacional de Alimentação e Nutrição (PNAN) apresentou implementação parcial em Fortaleza no período analisado. 

Apesar de mais de 25 anos de existência, não encontramos na literatura nenhum estudo de avaliabilidade da política, apenas de programas vinculados à ela ^19^^-^^21^, muito embora estudos desse tipo venham sendo realizados na área da saúde ^22^. 

O município não possui uma política própria de alimentação e de nutrição, tampouco o governo do estado. Ter uma política específica atenderia melhor às necessidades locais e promoveria uma descentralização mais efetiva ^23^^,^^24^. Assim, a análise do presente estudo se guiou pela política nacional. 

A análise documental revelou que a PNAN está normativamente bem consolidada a nível federal, o que significa um avanço institucional. Entretanto, a edição de normas legais não garante sua efetivação, o que requer vigilância para a sua execução ^25^.

A PNAN ocupa posição secundária em relação a outras políticas ^26^. A insuficiência de recursos financeiros provenientes do governo federal é apontada como limitante para responder às demandas da agenda de alimentação e de nutrição. Ao mesmo tempo, a gestão central reconhece a necessidade de maior aporte de recursos municipais com essa finalidade. Desafios para o financiamento das ações de alimentação e de nutrição já foram reportados ^4^. 

A implementação parcial dos componentes do modelo teórico-lógico tem repercussões na organização da atenção nutricional. A subutilização da vigilância alimentar e nutricional impossibilita o diagnóstico populacional, que é necessário para todas as fases do ciclo da vida ^23^^,^^27^. A vigilância alimentar e nutricional restrita aos titulares do Programa Bolsa Família indica uma visão burocrática das condicionalidades em saúde, exclusivamente para cumprimento das metas de gestão. O desconhecimento e a baixa valorização da vigilância alimentar e nutricional como componente do cuidado em saúde também foram encontrados em outro estudo ^28^. 

Embora tenham sido relatadas ações relativas aos programas de suplementação de micronutrientes, a cobertura é baixa, segundo relatórios públicos do Sistema de Vigilância Alimentar e Nutricional para Fortaleza ^29^, realidade recorrente em outros cenários nacionais ^30^^,^^31^. 

Apesar da relevância da promoção da alimentação adequada e saudável na PNAN, o cenário em Fortaleza é semelhante ao nacional, há priorização das ações de controle de agravos nutricionais em detrimento das ações de promoção da alimentação adequada e saudável ^4^^,^^32^. A publicação de guias alimentares com diretrizes nacionais representa um avanço nas ações de promoção da alimentação adequada e saudável, entretanto, ainda são necessárias estratégias de difusão das mensagens dos guias alimentares ^27^. 

O cuidado integral da pessoa com doenças crônicas e de necessidades nutricionais especiais é priorizado por meio de atendimentos individuais e domiciliares, retratando a persistência da hegemonia do modelo biomédico em saúde ^28^^,^^33^. A esse cenário, soma-se a sobrecarga de atividades das equipes e a falta de recursos materiais, o que impacta nas ações de promoção da saúde. 

Em relação às fragilidades de implementação da política em nível municipal, elas podem estar relacionadas à ausência de uma área técnica institucionalizada de alimentação e de nutrição no nível central para apoiar os técnicos das regionais de saúde de forma mais sistemática, conforme identificado em estudos com outros cenários ^24^^,^^34^. 

A intersetorialidade é vista tanto como fragilidade quanto potencialidade. Sua força em ações vinculadas ao Programa Saúde na Escola, ao Gente Adolescente e ao Programa Bolsa Família é percebida, porém, ela tem caráter pontual. Além da educação, são mencionadas experiências com a assistência social no escopo da segurança alimentar e nutricional, mas igualmente esporádicas. É importante que ocorra a ampliação da articulação intersetorial, para que a agenda de alimentação e de nutrição seja um tema presente na definição e no planejamento de outros setores ^35^, fortalecendo políticas públicas estruturantes e sinérgicas com foco nos principais agravos nutricionais ^20^.

Quanto ao impacto relacionado à política, a redução da magreza acentuada em crianças de 5 a 10 anos representa um reflexo da implementação da PNAN em Fortaleza, apesar da forte influência externa nesse desfecho.

Como limitações do estudo, salienta-se o fato de a validação do modelo teórico-lógico ter acontecido individualmente, não permitindo a negociação entre os diferentes informantes-chave sobre possíveis discordâncias. Contudo, a proposta de validação em oficina não foi acolhida pela gestão da Atenção Primária à Saúde devido a prejuízos relacionados à saída de profissionais dos serviços com alta demanda cotidiana. Ademais, o fato de o estudo ser proveniente de demanda acadêmica e não do serviço de saúde pode influenciar desdobramentos e avaliações futuras.

O estudo de avaliabilidade contribuiu para um melhor entendimento da PNAN em Fortaleza, desde os movimentos de institucionalização até a sua implementação no cotidiano. Além disso, permitiu envolver informantes-chave de diversos campos de atuação e elaborar o modelo teórico-lógico. 

Identificou-se diversos desafios na implementação da política. Entre eles, destaca-se a frágil apropriação dela pelos informantes-chave, apesar do desejo de fortalecê-la. Ressalta-se que os quatro componentes que compuseram o modelo teórico-lógico, representam, de maneira mais robusta, o alcance da implementação da política no município. 

Conclui-se que a PNAN é passível de avaliação, já que foi possível caracterizar seus elementos constituintes, oferecendo subsídios para uma avaliação de interesse dos participantes do estudo. São apresentadas as seguintes recomendações para a implementação da PNAN: investir na política em sua totalidade, não apenas em ações específicas e focadas em grupos prioritários; incluir mais indicadores da política nas pactuações do Sistema Único de Saúde e visibilizá-los para profissionais, gestores e controle social; revisar periodicamente o modelo teórico-lógico da política; divulgar os resultados desta pesquisa para gestores, técnicos, profissionais da estratégia saúde da família e para o controle social; realizar maior divulgação e formação sobre a política; fomentar financiamento municipal para a agenda de alimentação e de nutrição; propiciar maior inserção do nutricionista nas e-Multi; criar institucionalmente uma área técnica de alimentação e de nutrição com atribuições voltadas apenas a essa pasta e/ou com equipe técnica ampliada; fazer estudos avaliativos para todos os componentes descritos. 

O modelo teórico-lógico desenvolvido por esta pesquisa retrata um quadro que pode ser uma referência para ajudar os implicados na implementação da política, possibilitando revê-la por meio de um processo participativo ^10^. O modelo elaborado na presente pesquisa pode ser estudado e revisado a qualquer momento, a partir de novas diretrizes, de inovações em estratégias de implementação ou de outros olhares analíticos e/ou avaliativos.
